# Impact of maternal visceral leishmaniasis on sex-specific immune responses and pathogenesis in offspring following homologous infections

**DOI:** 10.3389/fimmu.2026.1667720

**Published:** 2026-01-29

**Authors:** Haruka Mizobuchi, Chizu Sanjoba, Yasuyuki Goto

**Affiliations:** Laboratory of Molecular Immunology, Department of Animal Resource Sciences, Graduate School of Agricultural and Life Sciences, The University of Tokyo, Tokyo, Japan

**Keywords:** developmental immune alterations, erythrophagocytosis, granulomatous inflammation, maternal infection, sex-specific immunity, visceral leishmaniasis

## Abstract

*Leishmania donovani* (Ld), the etiological agent of visceral leishmaniasis, has recently been implicated in vertical transmission, raising concerns about the potential impact of maternal infection on offspring immunity and disease susceptibility. Despite this, the effects of maternal Ld infection on the offspring’s immune responses and pathogenesis upon homologous infection remain largely uncharacterized. In this study, we investigated the influence of maternal Ld infection on disease outcomes in offspring by challenging offspring born to chronically infected female mice with homologous Ld parasites. Although persistent infection or acquired immune memory was not detected in offspring postnatally, distinct sex-dependent pathological outcomes were observed following challenge. Male offspring exhibited exacerbated erythrophagocytosis by splenic macrophages, leading to marked anemia irrespective of splenic parasite burden. In contrast, female offspring showed aggravated hepatic parasitic proliferation, inflammatory infiltration, granuloma formation, and extensive liver damage. These findings suggest that maternal Ld infection induces long-lasting, sex-specific alterations in the offspring’s immune system, particularly affecting macrophage function. This study provides the first evidence that maternal Ld infection differentially shapes the offspring’s immunopathological responses to homologous infection in a sex-dependent manner, offering novel insights for risk assessment and the development of sex-informed strategies for disease prevention.

## Introduction

Leishmaniasis is a parasitic disease caused by protozoan parasites of the genus *Leishmania*, which primarily infect host macrophages. Clinically, the disease is broadly classified into two major forms: visceral leishmaniasis (VL) and cutaneous leishmaniasis (CL). VL, primarily caused by *Leishmania donovani* (Ld) and *L. infantum*, presents with severe systemic symptoms such as fever, hepatosplenomegaly, and anemia, and can be fatal if left untreated. VL remains endemic in regions including South Asia, East Africa, and South America, where it continues to pose a significant public health concern (1).

Although *Leishmania* parasites are generally transmitted *via* the bite of infected female phlebotomine sandflies, increasing evidence from both animal models and human cases suggests the possibility of vertical (mother-to-fetus) transmission during pregnancy (2, 3). Moreover, independent of vertical transmission itself, maternal infection may influence the susceptibility and disease outcomes of offspring upon homologous infection, as has been demonstrated in other infectious disease models, such as helminth and *Trypanosoma* infections (4–6). These findings suggest that not only the pathogen, but also the maternal immune status and inflammatory environment, can exert long-term effects on the developing fetal and neonatal immune systems (7), raising the possibility of similar immunological mechanisms in leishmaniasis. If maternal Ld infection indeed alters offspring susceptibility or immune responses in the long term, this could have important epidemiological implications for VL-endemic areas, potentially affecting herd immunity in the next generation and influencing future transmission dynamics.

Despite this possibility, experimental studies examining how maternal *Leishmania* infection affects immune responses and disease development in offspring remain extremely limited. To our knowledge, only a handful of reports have addressed this issue. For example, Herman et al. reported that offspring born to Ld-infected female hamsters exhibited increased resistance to infection with the same parasite, suggesting potential immune priming due to maternal Ld infection (8). In contrast, Osorio et al. confirmed Ld vertical transmission in the same hamster model and found that Ld challenge infection of the offspring led to increased mortality and exacerbated pathology, indicating that maternal Ld infection may instead worsen VL outcomes in progeny (9). Similarly, Lima et al. demonstrated in a *L. braziliensis*-infected CL hamster model that offspring born to infected mothers developed more severe cutaneous lesions upon challenge infection (10).

Previous studies have relied exclusively on hamster models, which offer limited immunological tools and constrain detailed molecular analyses of host immune responses and organ pathology. Notably, studies employing mouse models—where immunological mechanisms can be investigated in greater detail—are virtually absent in the context of maternal-offspring immunity in leishmaniasis.

Recently, we established a pregnant VL mouse model and demonstrated, through genetic and histological analysis, the presence of Ld parasites in fetuses of chronically infected dams, providing clear evidence of vertical transmission (11). Building upon this model, the present study aimed to investigate how maternal VL influences the susceptibility and disease progression in offspring following homologous infection. To this end, we challenged offspring born to chronically Ld-infected mothers with the same parasite strain and examined disease outcomes.

## Materials and methods

### Ethics statement

The animal experiments were reviewed and approved by an institutional animal research committee and an institutional committee on genetically modified organisms at the Graduate School of Agricultural and Life Sciences, The University of Tokyo (Approval No. P22-117). The experiments were performed in accordance with the Regulations for Animal Care and Use of the University of Tokyo, which were based on the Law for the Humane Treatment and Management of Animals, Standards Relating to the Care and Management of Laboratory Animals and Relief of Pain (the Ministry of the Environment), Fundamental Guidelines for Proper Conduct of Animal Experiment and Related Activities in Academic Research Institutions (the Ministry of Education, Culture, Sports, Science and Technology) and the Guidelines for Proper Conduct of Animal Experiments (the Science Council of Japan).

### Experimental infection and mating

Female BALB/c mice at the age of 6 weeks were purchased from Japan Clea, Tokyo, Japan, and were acclimated for 1 week. All mice (3–5 mice per cage) were maintained under specific pathogen-free conditions in a temperature- and humidity-controlled room under a 12h light/dark cycle with unrestricted access to food and water. Ld parasites (MHOM/NP/03/D10; a gift from the National BioResource Project at Nagasaki University (12)) were cultured in medium 199 (Thermo Fisher Scientific, Waltham, MA, USA) supplemented with 10% heat-inactivated fetal bovine serum (Thermo Fisher Scientific), 100 U/ml penicillin and 100 μg/ml streptomycin (Wako, Osaka, Japan) at 25°C. Experimental infection was performed as previously described (13). Briefly, Ld parasites in late log or stationary phase were washed with PBS by centrifugation at 1,600 ×*g* for 10 min and were resuspended with PBS at the concentration of 1 × 10^8^ cells/ml. Female mice at the age of 7 weeks were infected with 1 × 10^7^ Ld parasites by intravenous injection into the tail vein. Thirty-two weeks after infection, the infected or naïve female mice were mated with healthy male mice (from Japan Clea) at one male per cage of two females over 2weeks. Female mice with a confirmed vaginal plug were transferred to individual cages. The pregnancy rates have been previously reported to be 86.7% in naïve dams and 53.8% in Ld-infected dams (11). In the present study, these established pregnancy rates were taken into account when designing and conducting the experiments. The offspring were weaned at 4 weeks of age, separated from their mothers, and housed by gender. For each experimental group, 5–6 dams were used, yielding 3–4 litters and a total of 17–21 offspring. The entire experiment was performed in three independent biological replicates. At each time point, offspring from different dams were pooled, and individual animals were randomly selected from this mixed pool for analysis. Thus, offspring from the same litter were not systematically treated as independent biological replicates, and no single litter disproportionately contributed to the dataset. This approach minimizes potential litter effects in the measurements. PCR assays targeting *Leishmania* parasite genes were performed on spleen and liver samples obtained from the 8-week-old offspring, as previously described (11).

For homologous Ld challenge infection experiments, offspring born to Ld-infected or naïve dams were challenged at 7 weeks of age with 1 × 10^7^ Ld parasites *via* intravenous injection into the tail vein. The offspring were monitored regularly for clinical signs and body weight. At 26 weeks post-infection, all offspring were euthanized for pathological and immunological analyses. For evaluation of parasite burden, stamp smears of the spleens and livers were fixed for 5 min in methanol and stained for 25 min with 5% Giemsa solution (Merck Millipore, Darmstadt, Germany). Amastigotes were counted by microscopic observation of the stained smear at 1,000× magnification, and Leishman-Donovan Units (LDU) were enumerated as the number of amastigotes per 1,000 host nuclei times the tissue weight in grams as performed in a previous study (13).

### Dissection and hematological analysis

Mice were euthanized by cardiac puncture under anesthesia with 4% isoflurane vapor (Pfizer Japan Inc., Tokyo, Japan), and their spleens and livers were collected for analysis. For hematological analysis, blood was collected using the heparinized capillary tubes (TERUMO, Tokyo, Japan) and hematocrit (Ht) was determined by centrifuging the tubes at 15,000 ×*g* for 10 min. For quantitative analyses of blood cells, the number of blood cells were counted by microscopic examination. The peripheral white blood cells (WBC) were counted with Türk’s solution (Merck Millipore). Serum was collected after centrifugation for 10 min at 2,000 ×*g*, and alanine aminotransferase (ALT) in serum were measured by Oriental Yeast Co., Ltd. (Tokyo, Japan) using the clinical chemical analyzer, BioMajestyTM JCA-BM6050 (JEOL Ltd., Tokyo, Japan). The concentration of serum IL-1β, TNF-α and IFN-γ were measured by using commercial sandwich ELISA kit (Thermo Fisher Scientific).

### IgG ELISA

Total IgG concentration in serum was measured using commercial sandwich ELISA kit (Thermo Fisher Scientific) according to the manufacturer’s protocol. Ld-specific IgG ELISA was performed as previously described (14), with additional details on assay sensitivity. Soluble *Leishmania* antigen (SLA) was prepared by sonication of Ld or *L. major* (Lm) parasites. SLA (1μg/well) was used for coating Nunc MaxiSorp 96-well plates (Thermo Fisher Scientific) for 4h at room temperature followed by overnight blocking at 4°C with PBS-T containing 1% BSA. Plates were washed 5 times with PBS-T and once with PBS and were incubated with serum samples (starting at 1:100, serially diluted 10-fold to 1:10^7^) in PBS-T with 0.1% BSA for 2h at room temperature. After washing, plates were incubated with HRP-conjugated goat anti-mouse IgG, IgG1 or IgG2a (1:2,000 in antibody dilution buffer; Southern Biotech, Birmingham, AL) for 1h. Color development was done using TMB substrate (Kirkegaard & Perry Laboratories, Gaithersburg, MD) for 2min, stopped with 1N H_2_SO_4_, and read at 450nm using a SpectraMax Paradigm reader (Molecular Devices, Sunnyvale, CA). Data were analyzed with SoftMax Pro 6 (Molecular Devices), and reciprocal endpoint titers were calculated in GraphPad Prism 10.2 (GraphPad Software Inc., San Diego, CA) using an OD cutoff of 0.12. The half-life of IgG was calculated using the one phase exponential decay model in GraphPad Prism 10.2 (GraphPad Software Inc.).

Analytical sensitivity (limit of detection, LOD) of the ELISA was determined as follows: the cut-off value was calculated as the mean OD of negative control sera plus 3 SD. Serial 10-fold dilutions of a high-titer positive serum were tested, and the LOD was defined as the highest serum dilution that yielded an OD value above the cut-off. In this assay, the LOD corresponded to a 1:10,000 serum dilution.

### Measurement of Ld-specific IFN-γ production

Spleens were collected from offspring mice at 8 weeks of age. Single-cell suspensions were prepared by mechanical dissociation followed by passage through a 70-μm cell strainer (Corning, Corning, NY). Red blood cells (RBC) were lysed using RBC lysis buffer (Merck Millipore), and the remaining splenocytes were washed and resuspended in RPMI 1640 medium (Wako) supplemented with 10% heat-inactivated fetal bovine serum (Thermo Fisher Scientific), 100U/mL penicillin, and 100μg/mL streptomycin (Wako). Splenocytes (5 × 10^6^ cells/ml) were cultured in 96-well culture plates in the presence of Ld-SLA (10 μg/mL), Lm-SLA (10 μg/mL) or concanavalin A (ConA, 3μg/mL, Wako) as a positive control. Unstimulated wells were included as negative controls. Cultures were maintained at 37°C in a humidified incubator with 5% CO_2_. After 72 hours of incubation, culture supernatants were collected and stored at −20°C until analysis. The concentration of IFN-γ in the supernatant was measured using a commercial sandwich ELISA kit (Thermo Fisher Scientific), according to the manufacturer’s instructions.

### Histopathology

Spleens and livers of offspring were fixed with 10% formalin neutral buffer solution (Wako) and embedded in paraffin. HE staining was performed as previously described (15). For the quantification of RBC-phagocytosing cells, HE-stained spleen sections (6 µm) were examined under a 1,000× magnification. RBC-phagocytosing cells and the number of engulfed RBCs per cell were counted in five randomly selected fields. For granuloma quantification, HE-stained liver sections were analyzed at 400× magnification. Granulomas and the inflammatory cells within each granuloma were counted in five randomly selected fields. Based on previous studies (16), granulomas were categorized into three types: (i) immature granulomas, consisting of infected Kupffer cells with clusters of ≤20 inflammatory cells; (ii) mature granulomas, composed of infected Kupffer cells and >20 clustered inflammatory cells; and (iii) clear granulomas, containing only small clusters of inflammatory cells without detectable amastigotes.

### Statistical analysis

Biological replicates were defined as individual animals. When technical replicates were performed (e.g., duplicate measurements of the same serum sample), they were averaged and counted as one value per animal. Statistical analyses were performed exclusively on biological replicates. All Statistical analyses were performed using GraphPad Prism 10.2 software package (GraphPad Software Inc.). Results are presented as mean ± standard error of the mean (SE). For comparisons at a single time point, two-way ANOVA with maternal infection and offspring sex as factors was used, followed by Sidak or Tukey multiple comparisons test. Student’s t test was used to compare the differences between two independent groups. For longitudinal measurements, including body weight and antibody kinetics, repeated measures two-way ANOVA was applied with maternal infection and offspring sex as between-subject factors and time as a within-subject factor. Normality and homogeneity of variance were confirmed using Shapiro-Wilk and Levene’s tests, respectively. *P* values less than 0.05 were considered significantly different. Potential outliers were assessed using predefined statistical criteria, including the ROUT method (Q = 1%) and the interquartile range (IQR) rule (values < Q1 − 1.5×IQR or > Q3 + 1.5×IQR). Each identified data point was carefully evaluated for biological plausibility. All points were considered biologically plausible, and no data were excluded. No arbitrary removal of data was performed. This procedure ensures transparency and reproducibility of our statistical analyses.

For body weight measurements, a larger sample size (n = 13–17 per group) was used because neonatal body weight is strongly influenced by litter size: pups from larger litters tend to have lower individual body weights, whereas pups from smaller litters tend to be heavier. Litter sizes did not differ significantly between naïve and Ld-infected dams (11), minimizing potential confounding effects. Furthermore, the influence of litter size on body weight gradually diminished with growth, and within-group variability in body weight decreased. Sample sizes for organ weights and immune parameters ranged from n = 3–6 per group. Prior to the study, sample size and power calculations were performed based on preliminary data and expected effect sizes. These calculations indicated that n ≥ 3 per group would provide >80% power to detect major differences in these parameters.

## Results

### No clinical VL symptoms in offspring born to Ld-infected dams

**Figure 1:**
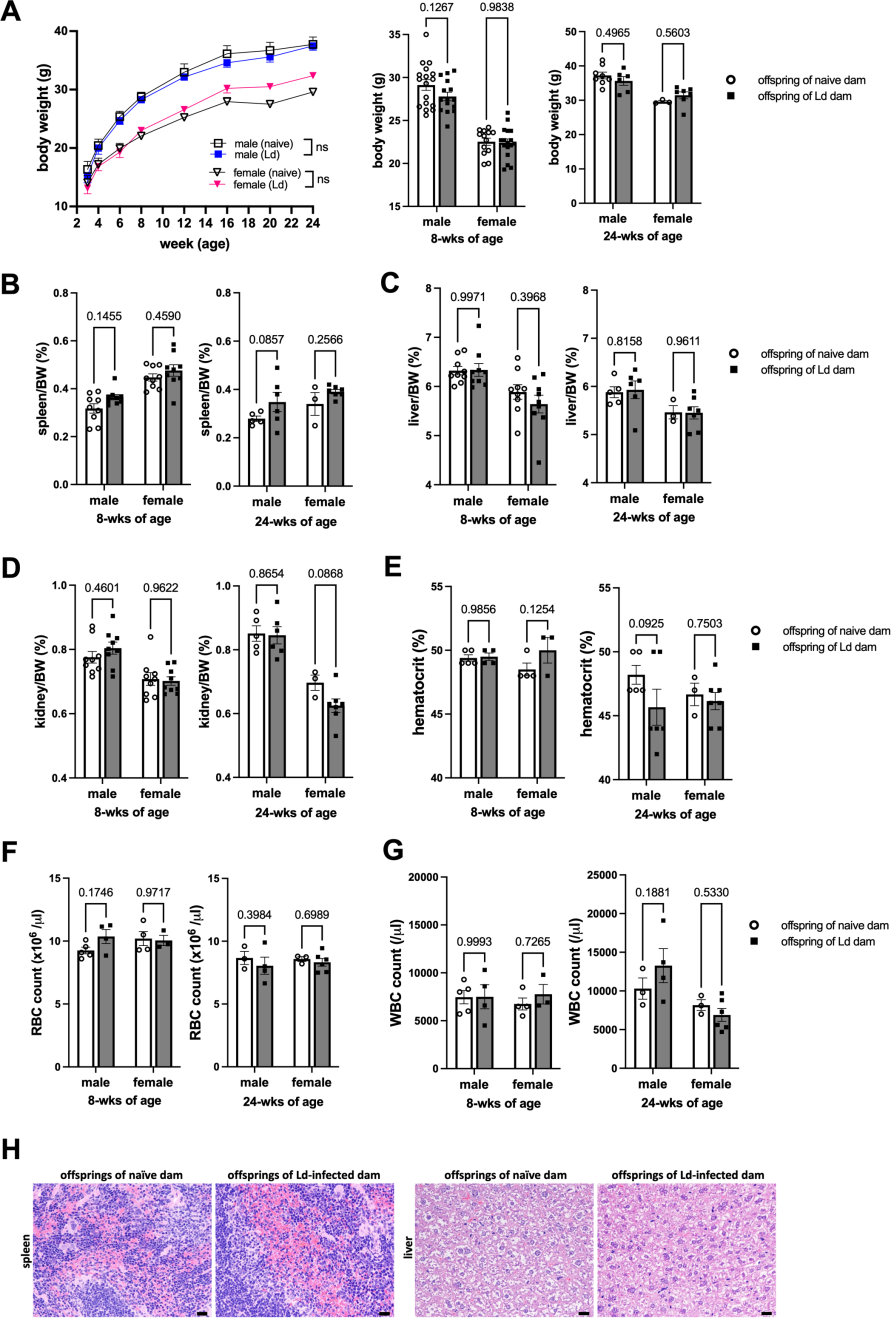
Absence of growth impairment and VL symptoms in offspring of Ld-infected dams. **(A)** Body weight change in offspring born to Ld-infected dams (8-wks of age: n = 13-17, 24-wks of age: n = 3-8). **(B–D)** Spleen, liver and kidney weights of 8- and 24-wks-old offspring (n = 3-9). **(E–G)** Peripheral blood hematocrit, RBC count, and WBC count of 8- and 24-wks-old offspring (n = 3-7). **(H)** Spleen and liver of 8-wks-old offspring. Bars, 20 μm. Means ± SE are presented. *P* values for two-way ANOVA with Sidak multiple comparisons are shown.

In our previous study, we reported that vertical transmission of Ld occurs in approximately 70% of fetuses in a pregnant Ld-infected mouse model (11). Based on this finding, we first investigated whether offspring born to Ld-infected mothers develop clinical signs of VL later in life. Longitudinal monitoring of body weight up to 24 weeks of age revealed that offspring from Ld-infected dams exhibited comparable growth to those born to uninfected controls (**Figure 1A**).

In our VL mouse model, infection with 1 × 10^7^ Ld parasites typically induces marked splenomegaly, hepatomegaly, and anemia by 24 weeks post-infection (13). However, at 24 weeks of age, the offspring of Ld-infected dams showed no significant changes in organ weights (**Figures 1B–D**) or hematological parameters (**Figures 1E–G**).

To assess the presence of Ld infection, PCR targeting the *Leishmania* large subunit ribosomal RNA gene was performed on spleen and liver tissues from 8-week-old offspring; however, all samples tested negative. Consistently, no parasites were observed in impression smears or histological sections of the spleen and liver. Histopathological examination at 8 weeks of age revealed no notable abnormalities, such as tissue degeneration or inflammatory infiltrates (**Figure 1H**). In addition, serum levels of pro-inflammatory cytokines, including IL-1β, TNF-α, and IFN-γ, were undetectable.

Taken together, these results indicate that, while vertical transmission of Ld parasites does occur in this pregnant VL mouse model, the offspring do not exhibit overt parasite burden or clinical symptoms of VL by 8 weeks of age.

### Maternal Ld infection does not induce Ld-specific adaptive immunity in offspring

**Figure 2:**
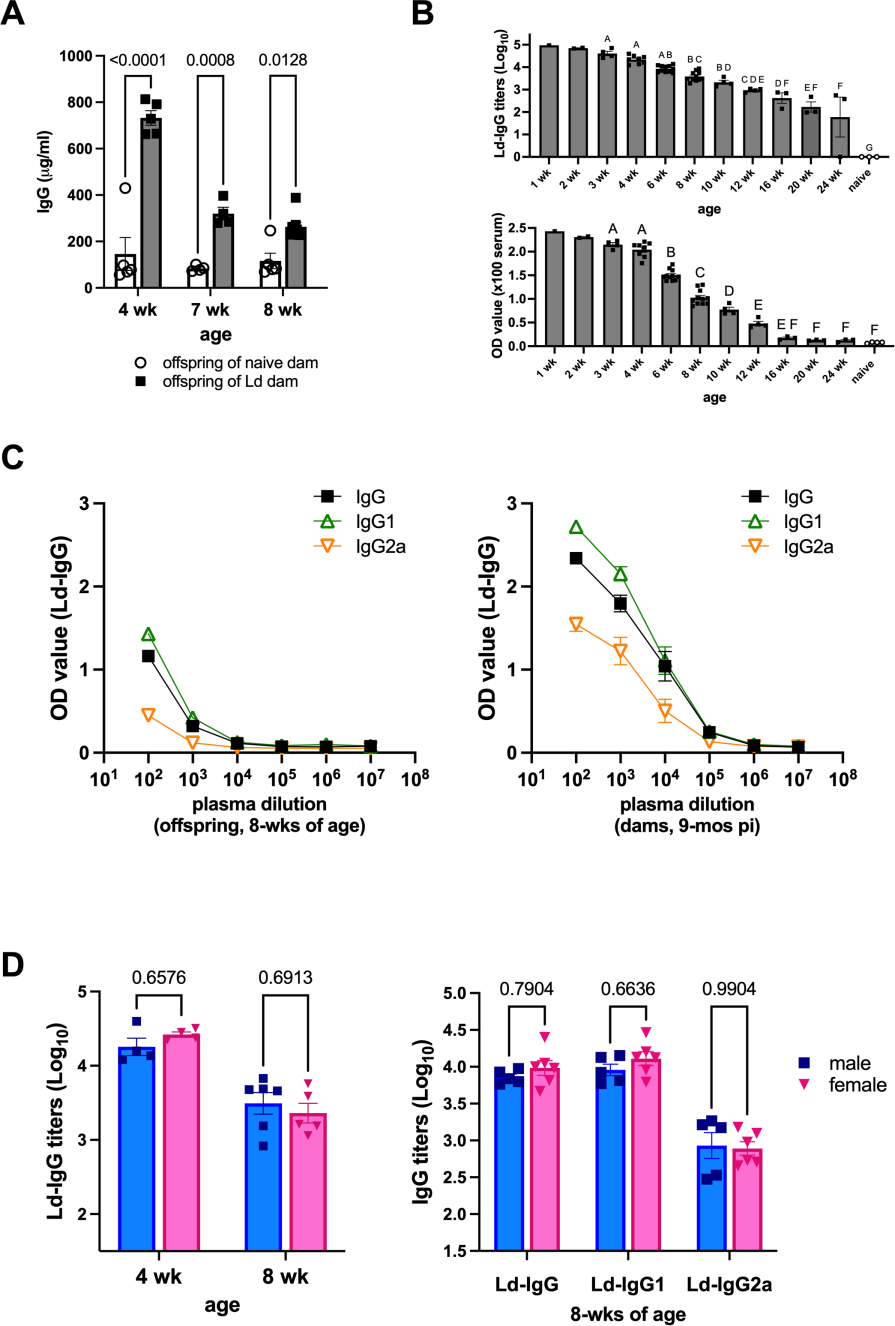
Age-dependent reduction of Ld-specific IgG in offspring of Ld-infected dams. **(A)** Serum total IgG concentration in offspring (n = 4-8). **(B)** Changes in Ld-specific IgG antibody titers (top) and the OD values of 100-fold diluted serum (bottom) in the offspring of Ld-infected dams (n = 1-11). Time points with n<3 (1 and 2 weeks) were plotted for descriptive purposes only and were excluded from statistical analyses; therefore, no significance letters are shown for these groups. **(C)** Comparison of Ld-specific IgG, IgG1, and IgG2a levels between 8-wks-old offspring (n = 11) and their dams at 9 months post Ld infection (n = 6). The OD values ​​of 100-fold diluted serum samples are shown. **(D)** Ld-specific IgG levels in male and female offspring (n = 4-6). Ld-specific IgG levels at 4 and 8-wks of age (left). Right: Ld-specific IgG, IgG1, and IgG2a levels at 8-wks of age (right). Means ± SE are presented. *P* values for one-way ANOVA with Tukey’s multiple comparisons or two-way ANOVA with Sidak multiple comparisons are shown. The uppercase letters in Figure 2 represent OD values (Optical Density).

Maternal transfer antibodies is a key component of neonatal immunity and, depending on the pathogen, can have either protective or detrimental effects on infection outcomes in offspring (17, 18). To examine the role of maternal antibodies in this context, we first analyzed the kinetics of serum antibodies in offspring born to Ld-infected dams. Total IgG levels in these offspring were significantly higher than in those from uninfected control dams (**Figure 2A**). This elevated IgG level declined markedly after weaning, although it remained higher than in control offspring.

We next assessed the levels of Ld-specific IgG. Similarly, Ld-specific IgG also decreased over time post-weaning and stabilized around 16 weeks of age (**Figure 2B**). The estimated half-life of Ld-specific IgG was 2.68 weeks. We further analyzed the subclass composition of Ld-specific IgG in 8-week-old offspring and their mothers (chronically infected for 9 months at the time of weaning). The IgG1/IgG2a ratio was comparable between dams and their offspring (**Figure 2C**), indicating that the Ld-specific antibodies detected in the offspring were primarily maternally derived. Notably, there was no significant sex-based difference in Ld-specific IgG levels among the offspring (**Figure 2D**).

**Figure 3:**
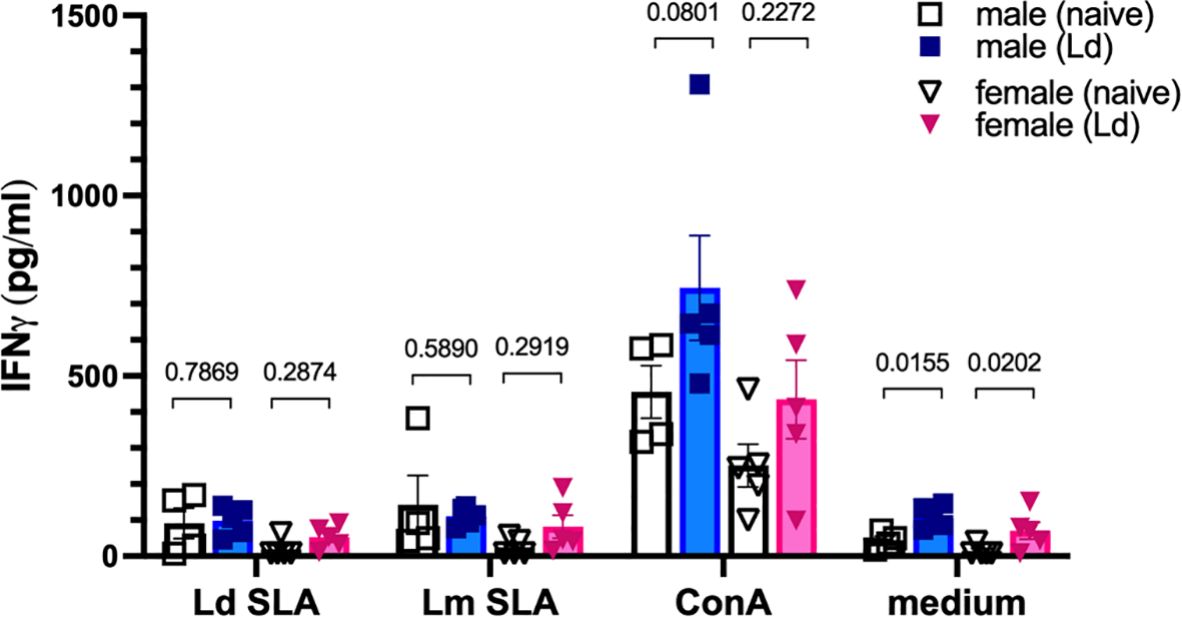
Maternal Ld infection does not induce Ld-specific adaptive immune response in offspring. IFNγ levels in the supernatants of offspring splenocytes stimulated with crude soluble antigen (SLA) of Ld for 72h. Splenocytes were harvested from 8-week-old uninfected offspring born to Ld-infected dams (n = 4-5). Ld SLA: *L. donovani* SLA (10 μg/ml), Lm SLA: *L. major* SLA (10 μg/ml), ConA: concanavalin A. (3 μg/ml). Means ± SE are presented. *P* values for two-way ANOVA with Sidak multiple comparisons are shown.

To assess whether the offspring developed Ld-specific adaptive immunity, splenocytes were isolated from 8-week-old offspring and stimulated *ex vivo* with Ld SLA for 72 hours, followed by measurement of IFN-γ in culture supernatants. Offspring born to Ld-infected dams failed to produce IFN-γ in response to Ld SLA, with levels comparable to both unstimulated cultures and those from control offspring (**Figure 3**). A similar lack of IFN-γ induction was observed when the splenocytes were stimulated with SLA from *Leishmania major*, indicating no cross-reactive T cell response. In contrast, stimulation with the mitogen Concanavalin A (ConA) resulted in robust IFN-γ production in all groups, confirming that general T cell responsiveness was intact. These findings demonstrate that although offspring from Ld-infected dams retain functional T cells, they do not exhibit Ld-specific T cell responses, suggesting that the circulating Ld-specific antibodies are passively acquired from the mother and not the result of endogenous immune priming.

### Maternal Ld infection exacerbates anemia in male offspring following homologous Ld challenge infection

To investigate the impact of maternal Ld infection on susceptibility and disease progression in offspring upon homologous challenge infection, 7-week-old offspring born to Ld-infected mothers were subjected to homologous Ld infection, and disease pathology was assessed 26 weeks post-infection. The results revealed that maternal Ld infection exerted sex-dependent effects on the offspring.

**Figure 4:**
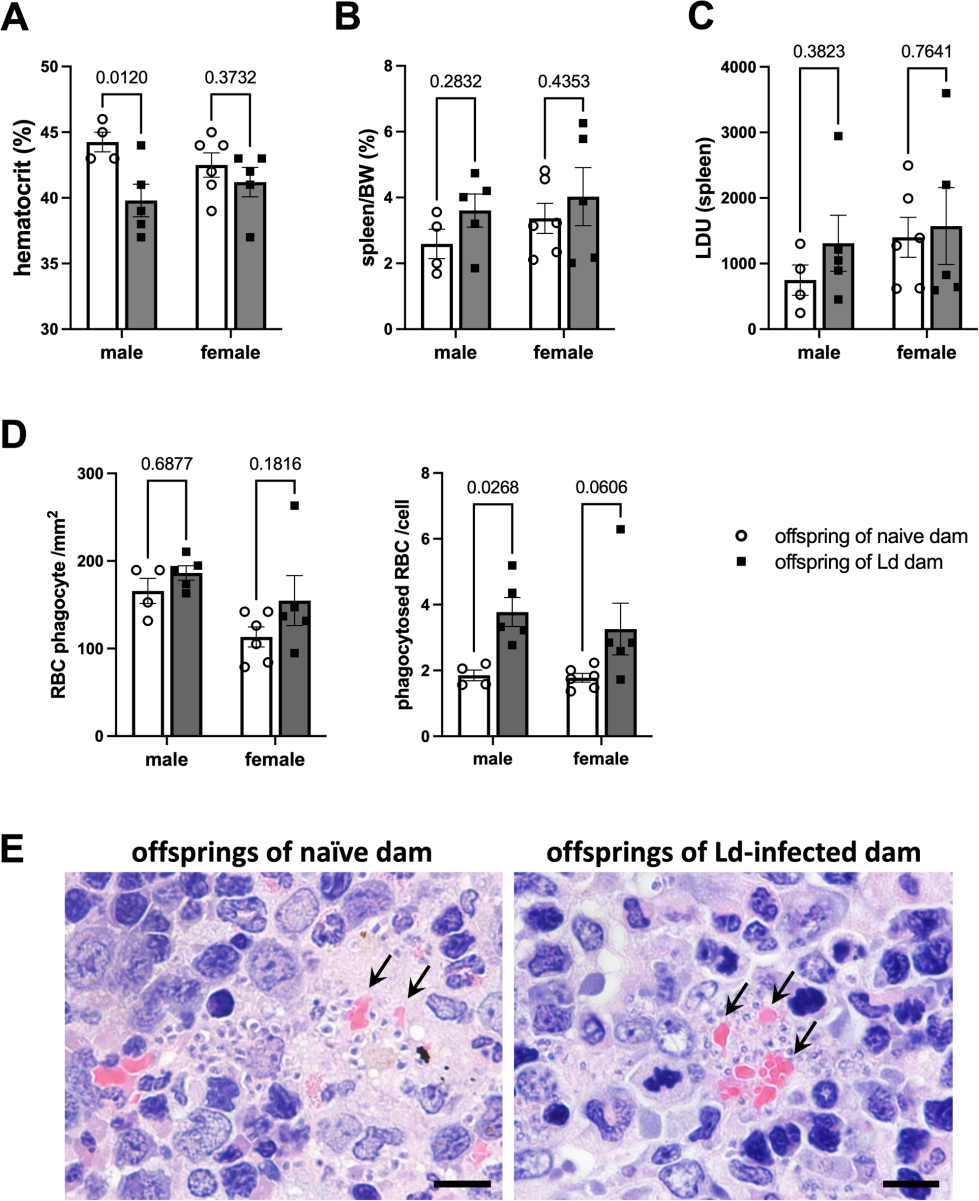
Maternal Ld infection exacerbates anemia in male offspring following homologous Ld challenge infection. Offspring born to Ld-infected dams were challenged with Ld at 7-wks of age, and VL-induced anemia was evaluated 26-wks post-infection (n = 4-6). **(A)** Peripheral blood hematocrit of Ld-infected offspring. **(B)** Spleen weight of Ld-infected offspring. **(C)** Leishman-Donovan Units (LDU) in the spleen of Ld-infected offspring. **(D)** Erythrophagocytic cells per unit area (left) and RBCs per phagocytic cell (right) in the spleen of Ld-infected offspring. The number of cells was counted in 5 random microscopic fields of HE-stained spleen section at 1000× magnification. **(E)** Representative erythrophagocytic cells in the spleen of Ld-infected male offspring. Arrow indicates the phagocytosed RBCs. Bars, 10 μm. Means ± SE are presented. *P* values for two-way ANOVA with Sidak multiple comparisons are shown.

In male offspring, a significant reduction in hematocrit levels was observed, indicating exacerbated anemia associated with Ld infection (**Figure 4A**). This finding suggests that maternal Ld infection aggravates VL-associated anemia in male offspring. In contrast, no significant differences were observed in spleen weight or splenic parasite burden (LDU), suggesting that the worsening anemia was not due to increased parasite load (**Figures 4B, C**).

Our previous studies have shown that erythrophagocytosis by infected macrophages in the spleen is related to anemia in this VL mouse model (13). Therefore, we performed quantitative analysis of erythrophagocytic cells in HE-stained spleen sections. While there was no significant change in the number of erythrophagocytic cells per unit area, the number of RBCs engulfed per cell was significantly increased (**Figures 4D, E**). In contrast, female offspring showed no significant changes in hematocrit levels (**Figure 4A**) and no increase in erythrophagocytic activity in the spleen (**Figure 4D**). Collectively, these results indicate that maternal Ld infection promotes RBC phagocytosis by splenic macrophages specifically in male offspring, thereby exacerbating anemia following homologous infection.

### Maternal Ld infection exacerbates hepatic granulomatous inflammation in female offspring following homologous Ld challenge infection

**Figure 5:**
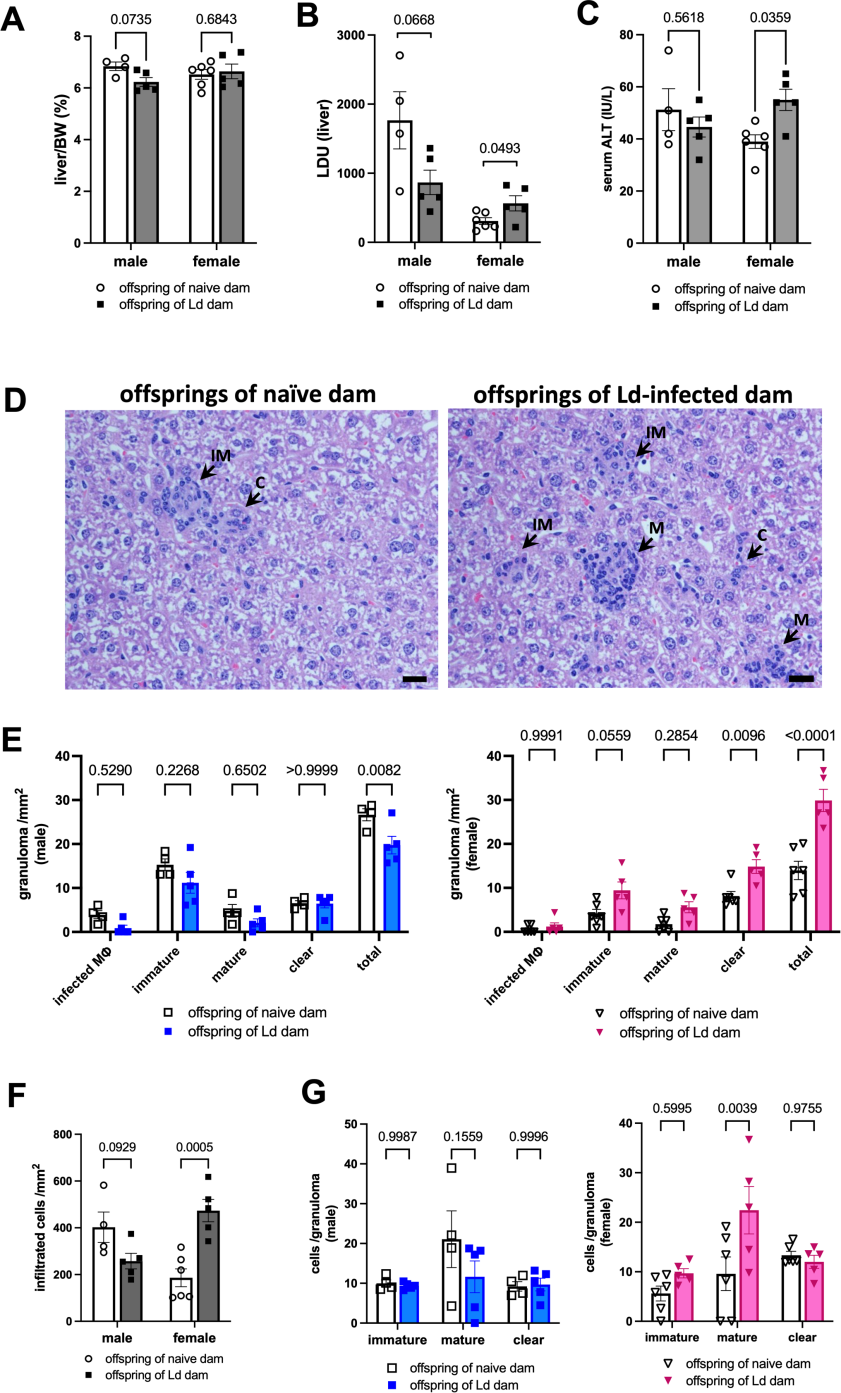
Maternal Ld infection exacerbates hepatic granulomatous inflammation in female offspring following homologous Ld challenge infection. Offspring born to Ld-infected dams were challenged with Ld at 7-wks of age, and VL-induced hepatic inflammation was evaluated 26-wks post-infection (n = 4-6). **(A)** Liver weight of Ld-infected offspring. **(B)** Leishman-Donovan Units (LDU) in the liver of Ld-infected offspring. **(C)** Serum ALT levels of Ld-infected offspring. **(D)** Representative granulomas in the liver of Ld-infected female offspring. IM: immature granuloma, M: mature granuloma, C: clear granuloma. Bars, 50 μm. **(E)** Classified granuloma counts per unit area in the liver of Ld-infected offspring. Infected MΦ: infected macrophages that are not surrounded by inflammatory cells. **(F)** Infiltrating cell density per unit area in the liver of Ld-infected offspring. **(G)** Inflammatory cell counts in each granuloma class in the liver of infected offspring. The number of cells was counted in 5 random microscopic fields of HE-stained liver section at 400× magnification. Means ± SE are presented. *P* values for two-way ANOVA with Sidak multiple comparisons are shown.

Next, we evaluated liver pathology induced by homologous Ld challenge infection in the offspring born to Ld-infected mothers. In contrast to the spleen, maternal Ld infection had a pronounced impact on liver pathology specifically in female, but not male, offspring (**Figure 5**). No significant changes in liver weight were observed in either sex (**Figure 5A**), indicating that maternal Ld infection did not affect hepatomegaly. However, female offspring born to Ld-infected mothers exhibited a significant increase in hepatic parasite burden (LDU) (**Figure 5B**), along with elevated serum ALT levels, a marker of liver dysfunction (**Figure 5C**).

Based on these findings, histopathological analysis was performed on HE-stained liver sections to evaluate granulomas and their constituent cells (**Figure 5D**). In female offspring of Ld-infected mothers, the number of hepatic granulomas was significantly increased (**Figure 5E**), accompanied by a higher degree of inflammatory cell infiltration (**Figure 5F**). Classification of granulomas based on cellular composition revealed a significant increase in “clear-type” granulomas—characterized by the absence of infected macrophages and composed mainly of inflammatory cells—in the female offspring of Ld-infected mothers (**Figure 5E**). There was also a trend toward increased numbers of “immature-type” granulomas, which consist of infected macrophages and fewer than 20 inflammatory cells. In contrast, the number of “mature-type” granulomas—those composed of infected macrophages surrounded by more than 20 inflammatory cells—as well as infected macrophages without inflammatory infiltration, showed no significant changes.

Importantly, the total number of granulomas, regardless of type, was significantly increased in female offspring of Ld-infected mothers (**Figure 5E**). Furthermore, quantitative analysis of inflammatory cell infiltration within each granuloma type revealed a marked increase in the number of infiltrating inflammatory cells specifically in mature-type granulomas in the female offspring (**Figures 5F, G**). These findings indicate that maternal Ld infection not only promotes increased granuloma formation but also contributes to granuloma enlargement due to enhanced cellular infiltration in female offspring.

In contrast, male offspring showed no significant changes in hepatic LDU or serum ALT levels (**Figures 5B, C**), and maternal Ld infection had no observable effect on granuloma number, inflammatory cell infiltration, or granuloma subtype distribution (**Figures 5E–G**). Notably, a slight reduction in the total number of granulomas was observed in male offspring born to Ld-infected mothers (**Figure 5E**).

Together, these findings demonstrate that maternal Ld infection exacerbates hepatic parasite proliferation and granulomatous inflammation following homologous infection specifically in female offspring. This study represents the first clear evidence that maternal Ld infection can exert long-term, sex-specific effects on chronic disease progression in the next generation.

## Discussion

In this study, we comprehensively analyzed the effects of maternal Ld infection on disease progression in offspring following homologous Ld challenge. Our results revealed that maternal Ld infection induces sex- and organ-specific pathological changes in the offspring. To date, research focusing on sex differences in the effects of maternal VL on offspring pathology has been extremely limited, and the underlying mechanisms remain largely unclear. Against this background, our study provides pioneering evidence highlighting the importance of considering sex differences in understanding the impact of maternal VL. Moreover, the mouse model used in this study offers advantages over traditional VL studies using hamsters, as mice have a uniform genetic background, making them suitable for analyzing sex differences and assessing the effects of maternal infection. Furthermore, a wide range of immunological tools, including antibodies and genetically modified mice, are readily available, allowing detailed investigation of immune responses and mechanistic studies. This enables more precise evaluation of sex-dependent immune responses and the long-term impact of maternal infection.

It should be noted that, due to inherent constraints of animal experiments, statistical power may be limited for some comparisons. Sample sizes were increased within ethical and practical limits whenever possible. Consequently, some non-significant results may reflect limited statistical power rather than a true absence of biological difference. This limitation should be considered when interpreting these findings.

Offspring born to Ld-infected mothers developed normally (**Figure 1**), and although vertical transmission of parasites was observed during the fetal period (11), no overt parasite burden was detected in the offspring postnatally, and no clinically detectable infection was observed at 8 weeks of age. The decline in Ld-specific antibodies during postnatal development (**Figure 2**) further supports the absence of ongoing parasitic replication in the offspring. Indeed, the parasite load transferred during vertical transmission—estimated to be on the order of 10^5^ (11)—was likely insufficient to induce overt VL pathology in the mouse model.

Moreover, offspring of Ld-infected mothers did not exhibit Ld-specific acquired immune responses (**Figure 3**). This aligns with previous reports showing that immune immaturity and tolerance during fetal and neonatal stages hinder the development of long-term immune memory against pathogens (19, 20), suggesting that vertically infected offspring fail to establish protective immunity against Ld.

Despite the lack of strong evidence for persistent Ld infection or Ld-specific immune memory, it is notable that maternal Ld infection significantly affected the offspring’s pathology following homologous challenge. These effects were sex-dependent and organ-specific, suggesting that maternal Ld infection can alter the offspring immune environment in a manner that differentially influences disease manifestations (7).

In male offspring, a significant decline in hematocrit levels was observed post-infection, indicating exacerbation of anemia (**Figure 4A**). However, this was not accompanied by changes in splenic parasite load or spleen weight (**Figures 4B, C**), suggesting that anemia severity was not linked to parasite burden. Histological analysis of spleen revealed no differences in the number of erythrophagocytic cells per tissue area, but an increased number of RBCs were phagocytosed per macrophage in male offspring (**Figures 4D, E**). These findings suggest that maternal Ld infection enhances erythrophagocytic activity of splenic macrophages in male offspring, which likely contributes to clinically significant anemia.

Our previous study showed that erythropoiesis is preserved in Ld-infected mice, as indicated by elevated erythropoietin levels and increased polychromatic erythrocytes (13), suggesting that reduced RBC production is unlikely. Furthermore, spleen size did not differ between sexes (**Figure 4B**), indicating that differential splenic sequestration of RBCs is also improbable. Taken together, enhanced erythrophagocytosis appears to be a major contributor to anemia in male offspring. The “don’t eat me” receptor SIRPα is a key molecule regulating hemophagocytosis, and our previous study in Ld-infected mice suggests that SIRPα is involved in macrophage-mediated erythrophagocytosis and anemia in this model (21, 22). Besides, previous studies have shown that the CD47–SIRPα signaling pathway is more prone to disruption in males, leading to excessive phagocytic activity (23). Based on these findings, it is conceivable that the SIRPα molecule may also be involved in the enhanced erythrophagocytic activity of macrophages observed in male offspring in this study; however, further validation is required.

On the other hand, female offspring also exhibited a similar tendency toward enhanced erythrophagocytosis (**Figure 4D**). However, the severity of anemia appeared to be relatively attenuated in females (**Figure 4A**). This sex difference may reflect sex-dependent physiological and immunological responses to *Leishmania* infection, and estrogen is known to promote iron absorption and improve anemia (24); however, further studies are needed to clarify the underlying mechanisms.

Having described the spleen-related findings, we next summarize the liver-related observations. In contrast, while no significant splenic pathology was observed in female offspring, hepatic granulomatous inflammation was markedly exacerbated (**Figure 5**). This included increased hepatic parasite burden (**Figure 5B**), elevated serum ALT levels (**Figure 5C**), enhanced granuloma formation (**Figure 5E**), and increased inflammatory cell infiltration (**Figures 5F, G**). The increase in “clear-type” granulomas—lacking infected macrophages and consisting solely of inflammatory cells—suggests an excessive immune response under conditions of poor parasite control. Moreover, in “mature-type” granulomas, the number of infiltrating inflammatory cells was also elevated (**Figure 5G**), indicating persistent chronic inflammation and aggravated hepatic tissue damage. Given that this female-specific hepatic pathology correlated with increased hepatic parasite burden, it is suggested that liver-resident macrophages (the host cells for Ld) underwent functional changes that promoted parasite proliferation. Indeed, maternal obesity has been reported to alter DNA methylation patterns in the livers of female but not male offspring, affecting hepatic metabolism over the long term (25). These observations suggest that functional changes of hepatic macrophages in female offspring, highlighting the importance of sex as a biological variable in disease pathogenesis.

Regarding sex differences in VL, epidemiological studies in humans suggest that males may have higher clinical VL incidence than females; however, findings are inconsistent depending on the region and study, and remain controversial (26). Although few studies exist in mouse models, males tend to have larger liver weights than females, which can be suppressed by exogenous estrogen administration (27). These observations are consistent with our findings of increased hepatic LDU and granulomas in male offspring, suggesting a potential role for sex hormones in VL pathology. Nonetheless, the relationship between sex hormones and VL pathology requires further investigation.

The sex-specific alterations in offspring pathology observed in this study may be driven by maternal immune modulation. Recent studies have demonstrated that maternal immune activation (MIA) due to infection or chronic inflammation can cause long-term, sex-specific changes in offspring immune function (7, 28, 29). The VL pathology observed in the offspring in this study may represent a novel example of MIA-induced modulation of the immune system. Furthermore, it has been reported that tissue-resident macrophages in the spleen and liver exhibit sex-specific immune responsiveness (30, 31). Taken together, our findings suggest that maternal Ld infection alters the offspring’s immune environment in a way that promotes organ-specific modulation of macrophage function, leading to sex-dependent manifestations of VL pathology in the offspring.

Because no sex-based differences were observed in the serum levels of maternally derived Ld-specific antibodies in the offspring (**Figure 2D**), it is unlikely that antibody levels account for the observed sex-specific VL pathology. However, studies in influenza virus infection models have reported sex differences in immune responses to maternal antibodies (32), and similar mechanisms cannot be excluded in our VL model. Notably, alterations in the maternal cytokine profile may exert long-term effects on the development of the offspring’s immune system (33, 34). Our previous work showed elevated IFN-γ levels in the serum of Ld-infected mothers, along with activation of IFN signaling pathways in placental tissues (11). Although these cytokine data are not presented again in the present study, these previous findings provide background evidence that maternal Ld infection could influence the offspring immune environment. In MIA models using Toxoplasma infection, maternal IFN-γ has been shown to alter hematopoietic stem cell function and differentiation in the fetus (35), suggesting that similar mechanisms might be occur in Ld infection. The sex-specific functional changes in macrophages observed here may reflect developmental biases in hematopoietic and immune cell lineages shaped by maternal IFN-γ signaling during gestation (29, 36). Taken together, these previous reports and the results of the present study suggest that the increase in IFN-γ associated with maternal Ld infection could influence the development of immune cells in the offspring hematopoietic system, leading to long-lasting changes in macrophage function after birth.

Thus, inflammatory cytokines, including IFN-γ, induced by maternal Ld infection may cause long-lasting “maternal infection–linked immune alterations” in the development of the offspring’s immune system and macrophage lineages, which could influence disease outcomes upon subsequent infection. The formation of sex-dependent VL pathology is likely driven by complex interactions between maternal–offspring cytokine signaling and immune system development. In addition to evaluating maternally derived antibodies, future studies should investigate the role of maternal cytokines in shaping offspring immune development in more detail.

In conclusion, these results suggest that maternal Ld infection alters the functional properties of offspring macrophages. These changes may have long-term and sex-specific effects on VL pathology following homologous Ld infection in the offspring. This study represents one of the first reports demonstrating the association between maternal infection and alterations in the offspring immune system in a VL model, providing important insights into immune regulation, including sex-specific effects. These results may help inform future risk assessment and preventive strategies that consider the sex of offspring in the context of maternal infection.

As limitations of the current study and future directions, mechanistic investigations—such as epigenetic and transcriptomic analyses of purified macrophages—will be necessary to directly evaluate the presence and nature of any functional “reprogramming.” Further studies should also elucidate the molecular mechanisms underlying immune modulation in offspring, including the roles of specific immune cell populations, cytokine networks, SIRPα signaling, sex hormone–mediated pathways, epigenetic modifications, and the transmission of maternal immune factors via the placenta or breast milk.

## Data Availability

The original contributions presented in the study are included in the article/supplementary material. Further inquiries can be directed to the corresponding author.
